# Identification and validation of the role of c-Myc in head and neck squamous cell carcinoma

**DOI:** 10.3389/fonc.2022.820587

**Published:** 2022-08-31

**Authors:** Sufeng Zhao, Li An, Xudong Yang, Zheng Wei, He Zhang, Yufeng Wang

**Affiliations:** ^1^ Department of Oral and Maxillofacial Surgery, Nanjing Stomatological Hospital, Medical School of Nanjing University, Nanjing, China; ^2^ Department of Geriatrics, Zhongda Hospital Southeast University, Nanjing, China; ^3^ Department of Pediatric Dentistry, Nanjing Stomatological Hospital, Medical School of Nanjing University, Nanjing, China; ^4^ Department of Periodontology, Nanjing Stomatological Hospital, Medical School of Nanjing University, Nanjing, China

**Keywords:** head and neck squamous cell carcinoma, c-Myc, survival, mRNA, triptonide

## Abstract

**Background:**

Many studies have shown that c-Myc plays a critical role in tumorigenesis. However, the molecular role of c-Myc in head and neck squamous cell carcinoma (HNSC) remains unclear.

**Methods:**

Several biological databases, including UALCAN, TIMER2.0, TCGAportal, GEPIA, KM plotter, OncoLnc, LinkedOmics, GSCA, and TCIA, were used to analyze the molecular role of c-Myc in HNSC. The expression levels of c-Myc were validated by real-time PCR (RT–PCR) and Western blot in CAL-27 cells.

**Results:**

The expression of c-Myc mRNA were significantly increased in HPV-negative HNSC tissues. The expression of c-Myc gene level was correlated with TP53 mutation status. HNSC also showed hypomethylated c-Myc compared with normal tissues. c-Myc was identified as an ominous prognostic factor for HNSC patients and correlated with immune infiltrating levels. Moreover, high c-Myc expression was associated with decreased expression of a series of immune checkpoints, resulting in a dampened immune response. c-Myc potentially mediated IL-17 signaling pathway and Th1 and Th2 cell differentiation. Inhibition of c-Myc expression increased apoptosis of CAL-27 cells.

**Conclusions:**

These findings suggest a new mechanism of c-Myc in the prognosis of HNSC, implying the potential of c-Myc as a therapeutic target for HNSC patients.

## Introduction

Head and neck squamous cell carcinoma (HNSC) is the eighth most frequent tumor worldwide and is associated with a high rate of morbidity and mortality (1). Despite great advances in diagnostic and therapeutic methods, a high rate of local and distant failure after treatment of advanced HNSC was observed (2). The prognosis for these patients with recurrent and metastatic (R/M) HNSC receiving platinum-based therapy is even worse (3). Hence, there is a high need for improved therapy for this population. A variety of genomic imbalances are involved in head and neck carcinogenetic processes (4–6).

The c-Myc proto-oncogene located at 8q24 has been implicated in the regulation of cell growth, differentiation and apoptosis (7). c-Myc overactivation is a frequently detected and crucial genetic event in HNSC. c-Myc gene amplification is involved in the pathogenesis of HNSC, especially in laryngeal squamous cell carcinoma (LSCC), which is the prominent histopathological entity among HNSC (8). Significant c-Myc amplification detected by implementing the polymerase chain reaction technique was observed in another study (9). c-Myc promotes the activation of poly (ADP-ribose) polymerase (PARP)-dependent DNA repair pathways, resulting in chemoresistance (10). c-Myc also promotes CHK1 and CHK2 expression to mediate the DNA damage checkpoint response, resulting in radioresistance (11). Overactivation of c-Myc seems to be correlated with aggressive biological behavior in HNSC, so its detailed mechanisms necessitate further study. The purpose of our study was to investigate the molecular role of c-Myc in HNSC to provide a new effective treatment option for HNSC patients.

## Materials and methods

### Expression and methylation of c-Myc in HNSC

UALCAN (12) and TIMER2.0 (13) were applied to evaluate the expression c-Myc in HNSC patients. The comparison of c-Myc expression between HNSC and normal samples was conducted using TCGA-HNSC dataset. The expression of c-Myc in subgroups of HNSC was subdivided based on TP 53 mutation status.

### Survival analysis of c-Myc in HNSC

TCGAportal (http://www.tcgaportal.org), GEPIA (14), KM plotter (15), and OncoLnc (16) were used to evaluate the impact of c-Myc mRNA expression on OS in HNSC patients based on the TCGA database.

### Relationships between c-Myc and immune checkpoints in HNSC

The relationships between c-Myc expression and immune checkpoints in HNSC patients from TCGA database were further investigated *via* LinkedOmics (17).

### Immune infiltration analysis of c-Myc in HNSC

GSCA (18) was used to detect the correlation between c-Myc expression and the immune microenvironment in HNSC. TIMER2.0 (13) and TCIA (19) were used to estimate the impact of immune cells on the OS of HNSC patients.

### Explore c-Myc related pathways in HNSC

Kyoto Encyclopedia of Genes and Genomes (KEGG) pathway enrichment analyses were conducted to understand the functions of c-Myc *via* LinkedOmics (17).

### Materials and cell culture conditions

Triptonide (purity > 98%) was purchased from Sigma (St. Louis, USA) and dissolved in dimethyl sulfoxide (DMSO) as a 5 mM stock and then freshly diluted in culture media at 10 nM, 30 nM, and 50 nM. Oral adenosquamous carcinoma cells, CAL-27 cells, were obtained from the Chinese Academy of Sciences Cell Bank (Shanghai, China). CAL-27 cells were cultured in RPMI-1640 supplemented with 10% fetal bovine serum in a humidified atmosphere containing 5% CO2 at 37°C.

### c-Myc siRNA

Synthetic c-Myc siRNA was used to knockdown the expression of c-Myc in CAL-27 cells. c-Myc siRNA (target sequence: 5′- GAGGAUAUCUGGAAGAAAUTT -3′; antisense:5′- AUUUCUUCCAGAUAUCCUCTT -3′) was purchased from Kaiji (Nanjing, China). Lipofectamine RNAiMax was purchased from Invitrogen (Grand Island, USA). Transfection was performed according to the manufacturer’s instructions. Human non-specific siRNA ((Kaiji, Nanjing, China)) was used as a negative control (NC).

### Apoptosis analysis

Annexin V/propidium iodide (PI) apoptosis detection kit (Biyuntian, Shanghai, China) was used to detect cell apoptosis according to the manufacturer’s protocol. CAL-27 cells were exposed to different concentrations of triptonide (10 nM, 30 nM, or 50 nM) for 72 h and then collected. Cells were resuspended in 200 μL binding buffer containing 5 μL Annexin V and 10 μL PI fluorescence dye, and then apoptosis was detected by flow cytometry (Becton Dickinson FACS Calibur).

### Western blot

Total proteins were extracted using SDS lysis buffer (Beyotime, Shanghai, China) supplemented with protease and phosphatase inhibitor cocktail (Beyotime). Protein samples were subjected to 12% SDS–PAGE and then transferred to PVDF membranes (Bio–Rad, California, USA). PVDF membranes containing proteins were blocked by QuickBlock Blocking Buffer at room temperature for 20 min and then incubated overnight at 4 °C with primary antibody. After washing, the blots were soaked with horseradish peroxidase (HRP)-conjugated secondary antibody at room temperature for 1 h. Finally, blots were visualized using Immobilon Western Chemiluminescent HRP Substrate (Millipore).

### Real-time PCR

Total RNA was extracted using the RNA-Quick Purification Kit (Esunbio, Hangzhou, China) and then used in a reverse transcriptase reaction with the PrimeScript™ RT reagent kit (Takara, Kyoto, Japan). The TB Green™ Premix Ex Taq™ kit (Takara) was used for the thermocycling reaction in an ABI-7500 fast Real Time PCR machine (Thermo Scientific). The primer sequences used in the experiment were as follows: c-Myc (Forward: 5′-CGCCAGAGGAGGAACGAGCTAA-3′; Reverse: 5′- TCTGCTTGGACGGACAGGATGT- 3′).

### Human oral squamous cell carcinoma tissues

Twenty of the oral squamous cell carcinoma (OSCC) tissue samples were obtained from the tissue bank of the department of pathology, Nanjing Stomatological Hospital. The study was approved by the institutional review board of the hospital. Informed consent was collected from all patients or their direct relatives. All diagnoses were histologically confirmed.

### Tissue microarrays (TMA) construction and immunohistochemistry (IHC)

Protein expression of c-Myc, PDCD1 and LAG3 was analyzed by IHC using a tissue microarray (TMA) platform. The TMA was constructed using pretreatment formalin-fixed paraffin-embedded (FFPE) tumor specimens from 20 adult patients with OSCC. The samples included 11 (55.0%) males and 9 (45.0%) females, with the mean age of 62.0 years old (range: 23-77 years old). Sections (5 μm) were then cut from each TMA and stained with antibodies to c-Myc (1:400; Abcam, Cambridge, USA), PDCD1 (1:200; Abcam, Cambridge, USA), and LAG3 (1:500; Abcam, Cambridge, USA). IHC staining was performed as previously reported (20, 21).

### Statistical analysis

Student’s t test, χ2 test and one-way ANOVA were used for comparisons, as appropriate. The difference was considered statistically significant if P<0.05. All analyses were performed using GraphPad Prism 8 (GraphPad Software, California, USA).

## Results

### c-Myc is significantly differentially expressed between TP53 wild-Type and mutant HNSC

**Figure 1:**
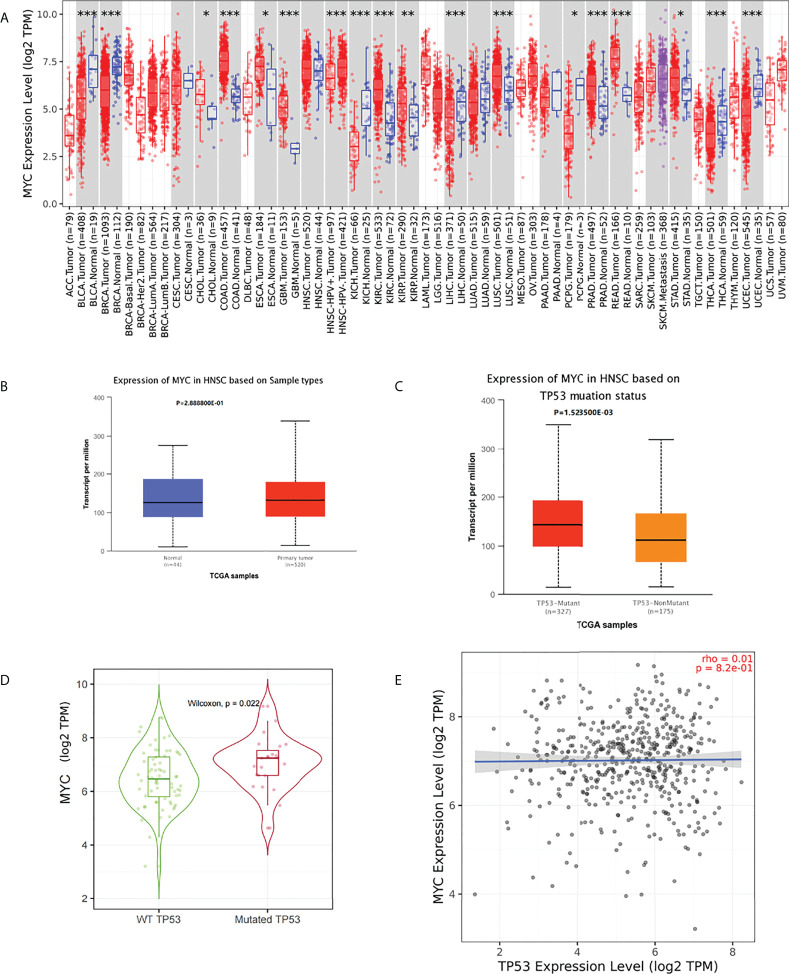
**(A)** c-Myc mRNA expression in pancancer (*: p-value < 0.05; **: p-value <0.01; ***: p-value <0.001). **(B)** c-Myc mRNA expression in HNSC (UALCAN). **(C)** c-Myc mRNA expression based on TP 53 mutation status (UALCAN). **(D)** c-Myc mRNA expression based on TP 53 mutation status (TIMER2.0). **(E)** Correlation between MYC and TP53 expression (TIMER2.0Fig 1E).

No significant difference in c-Myc mRNA expression in HNSCs was observed (**Figures 1A, B**). However, patients with HPV-negative HNSC had significantly higher c-Myc mRNA expression (**Figure 1A**). c-Myc expression was correlated with the mutation status of TP53 (**Figures 1C, D**). There was no correlation between c-Myc and TP53 expression (**Figure 1E**).

### c-Myc is a prognostic biomarker in HNSC

**Figure 2:**
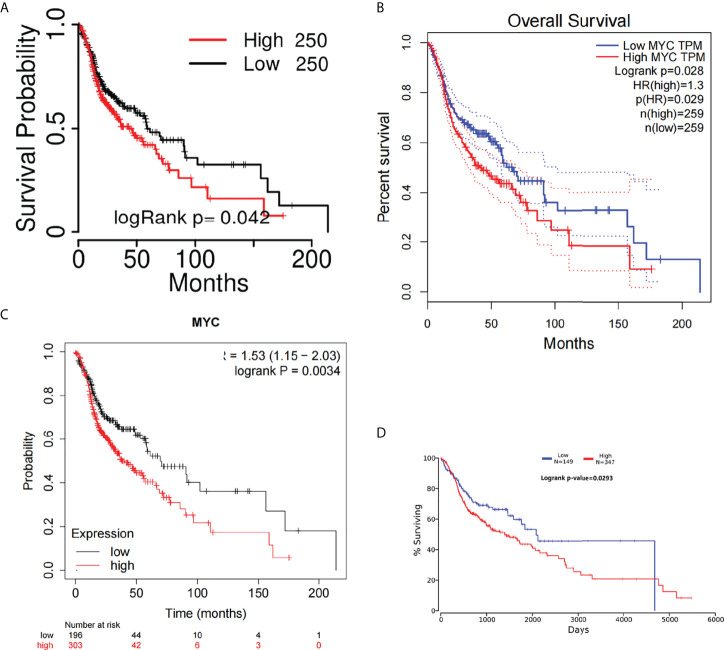
Overall survival curves of c-Myc in HNSC from different databases. **(A)** TCGAportal. **(B)** GEPIA. **(C)** KM-plotter. **(D)** OncoLnc.

**Figure 3:**
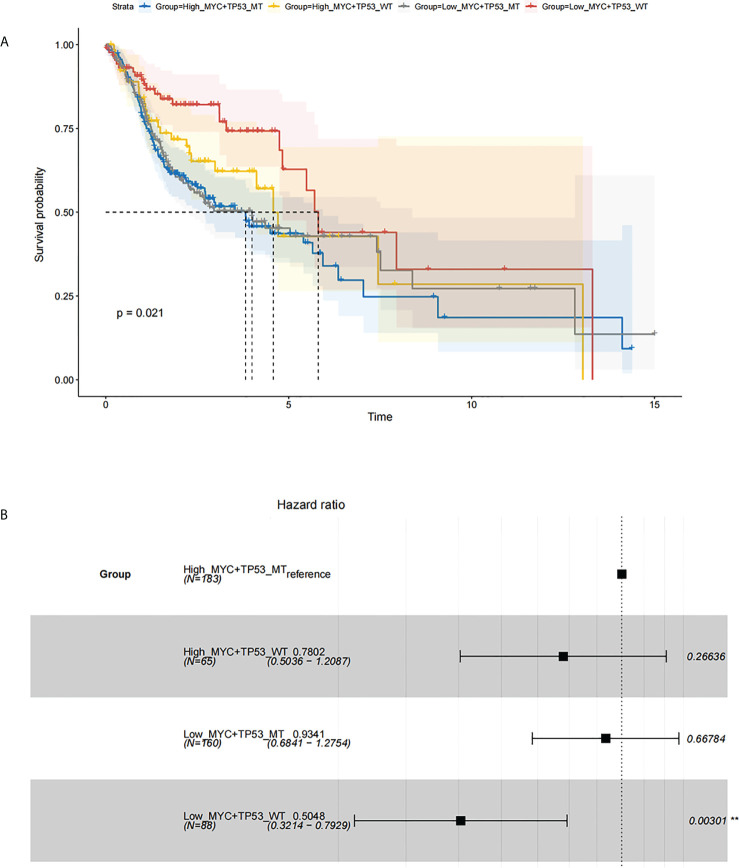
Survival analysis based on c-Myc expression and TP53 mutation status. **(A)** Kaplan Meier model. **(B)**. Cox proportional hazards model.

The overall survival (OS) of HNSC patients with low c-Myc mRNA expression was superior to that of patients with high c-Myc mRNA expression (**Figures 2A–D**). The cutoff values of c-Myc mRNA expression were different in these databases. These results revealed that c-Myc mRNA is a valuable prognostic biomarker in HNSC. TP53 mutant significantly deteriorated the survival of HNSC patients with low c-Myc mRNA expression (**Figures 3A, B**).

### c-Myc expression is related to immune checkpoint (ICP) genes in HNSC

**Figure 4:**
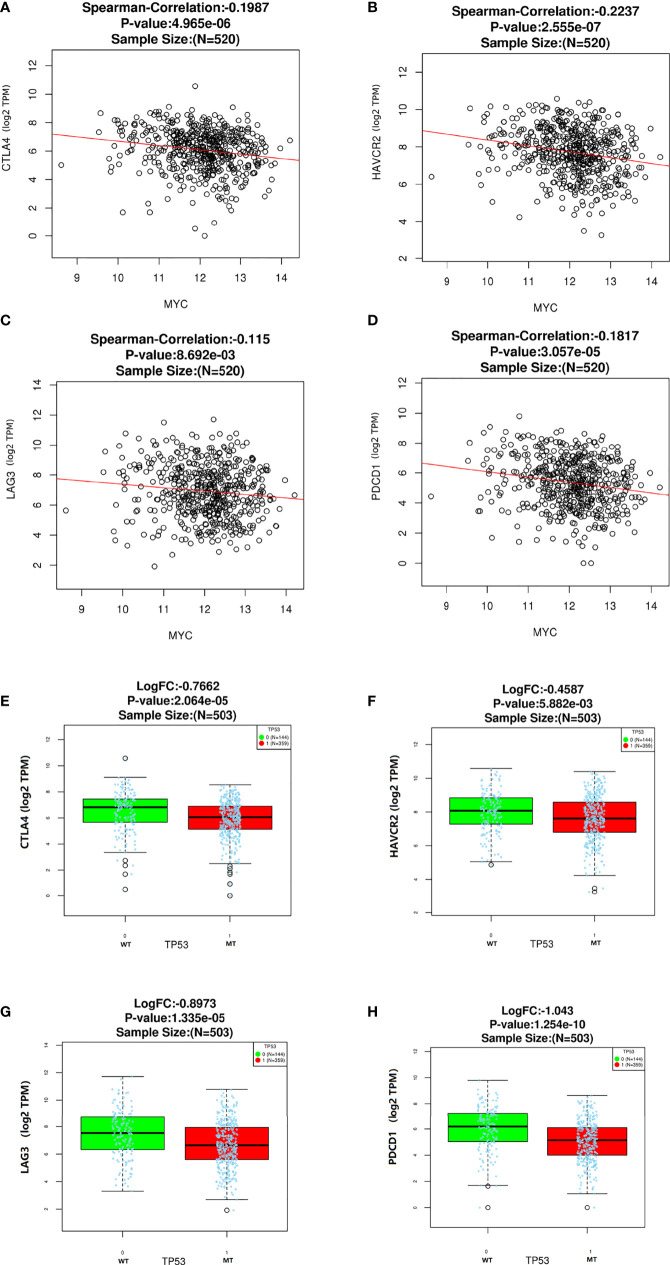
Correlation between c-Myc and immune checkpoints. **(A)** CTLA4. **(B)** HAVCR2. **(C)** LAG3. **(D)**Immune checkpoints expression based on TP 53 mutation status. **(E)** CTLA4. **(F)** HAVCR2. **(G)** LAG3. **(H)** PDCD1.

In this study, we analyzed the association of c-Myc expression and TP53 mutation status with immune checkpoints in the TCGA database. As shown in **Figure 4**, some inhibitory checkpoint molecules, including CTLA4, HAVCR2, LAG3 and PDCD1, had negative correlations with c-Myc mRNA levels in HNSCs. HNSC patients with TP53 mutant had low expression of CTLA4, HAVCR2, LAG3 and PDCD1 (**Figures 4E–H**).

### c-Myc expression is correlated with immune cell infiltration in HNSC

**Figure 5:**
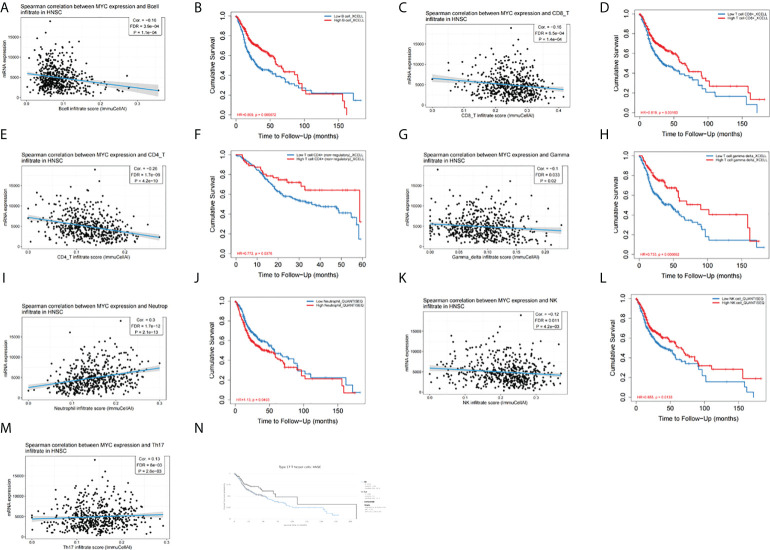
c-Myc expression is correlated with immune infiltration of **(A)** B cells, **(C)** CD8+ T cells, **(E)** CD4+ T cells, **(G)** gamma delta T cells, **(I)** neutrophils, **(K)** NK cells, and **(M)** Th17 cells. Tumor-infiltrating immune cells were associated with OS **(B, D, F, H, J, L, N)**.

**Figure 6:**
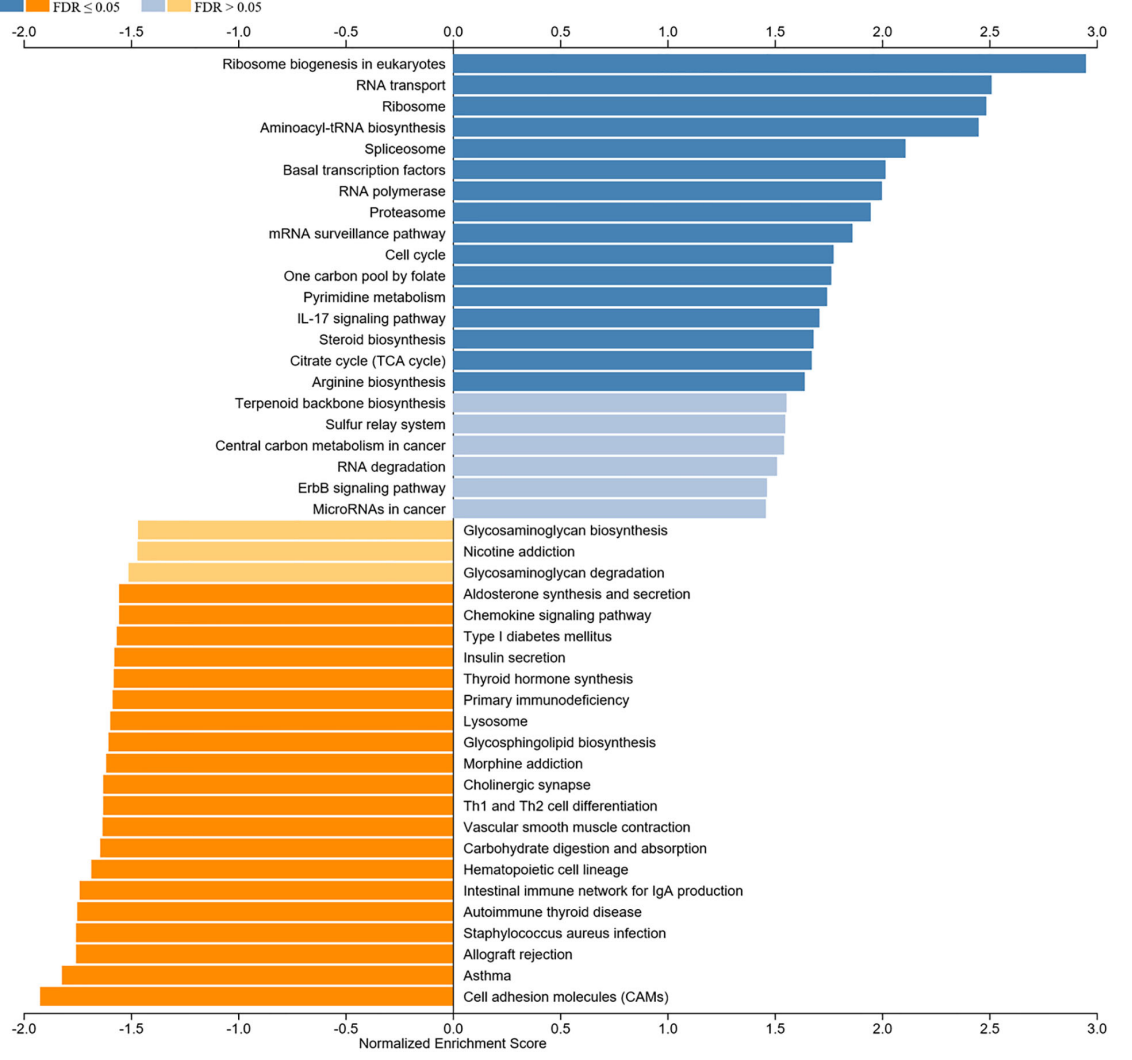
KEGG analysis of c-Myc.

We aimed to explore the correlation of c-Myc expression with immune cell infiltration in HNSC. The expression of c-Myc was negatively correlated with the infiltration of B cells, CD8+ T cells, CD4+ T cells, gamma delta T cells, and NK cells in HNSC patients (**Figures 5A, C, E, G, K**). In contrast, c-Myc expression had a positive correlation with the enrichment of neutrophils and Th17 cells (**Figures 5I, M**). As expected, the association of these tumor-infiltrating immune cells with OS was also observed in HNSC (**Figures 5B, D, F, H, J, L, N**). LinkedOmics was used to construct KEGG pathway enrichment analyses (**Figure 6**). c-Myc potentially mediated IL-17 signaling pathway and Th1 and Th2 cell differentiation to affect immune cell infiltration.

### Triptonide induces apoptosis of CAL-27 cells *via* c-Myc signaling

**Figure 7:**
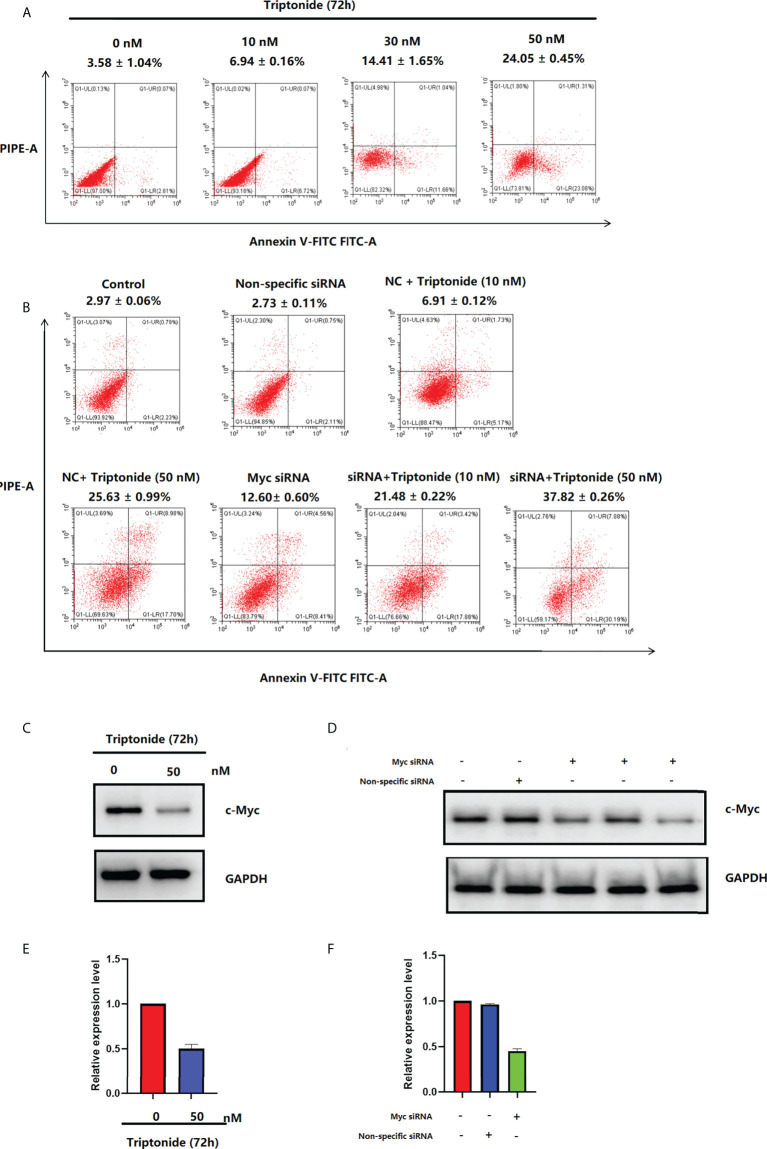
**(A)** Triptonide induces cell apoptosis. **(B)** c-Myc siRNA transfection induces cell apoptosis. **(C)** c-Myc levels in CAL-27 cells after treatment with triptonide, assessed by western blot. **(D)** The expression of c-Myc measured by western blot after c-Myc siRNA transfection. **(E)** c-Myc levels in CAL-27 cells after treatment with triptonide, assessed by RT–PCR. **(F)** The expression of c-Myc measured by RT–PCR after c-Myc siRNA transfection.

The apoptosis ratio of CAL-27 cells was investigated using Annexin V-FITC/PI staining and flow cytometry (**Figure 7A**). After incubation with 50 nM triptonide for 72 h, the percentage of apoptotic cells was 24.05 ± 0.45% (P < 0.01). Interestingly, low-dose (10 nM) triptonide treatment for 72 h also resulted in obvious apoptosis of CAL-27 cells. The percentage of apoptotic cells increased from 3.58 ± 1.04% to 6.94 ± 0.16% (P < 0.01) at 10 nM for 72 h. c-Myc specific siRNA also significantly increased the percentage of apoptotic cells. On the basis of triptonide treatment, we added c-Myc-specific siRNA to knockdown c-Myc expression (**Figures 7D, F**) and observed that the percentage of apoptotic cells still increased significantly (**Figure 7B**). We treated CAL-27 cells with 10 nM triptonide for 72 h and then collected cells for analysis of c-Myc protein levels using Western blotting. We observed that triptonide treatment and c-Myc specific siRNA induced a decrease in c-Myc protein (**Figures 7C, D**). To reveal the mechanism by which triptonide reduces c-Myc proteins, we tested the c-Myc mRNA level in CAL-27 cells (**Figure 7E**) and observed that triptonide treatment led to a decrease in c-My, suggesting that trip¬tonide regulates c-Myc at the transcriptional level.

### LAG3 expression tended to increase in patients with low expression of c-Myc

**Figure 8:**
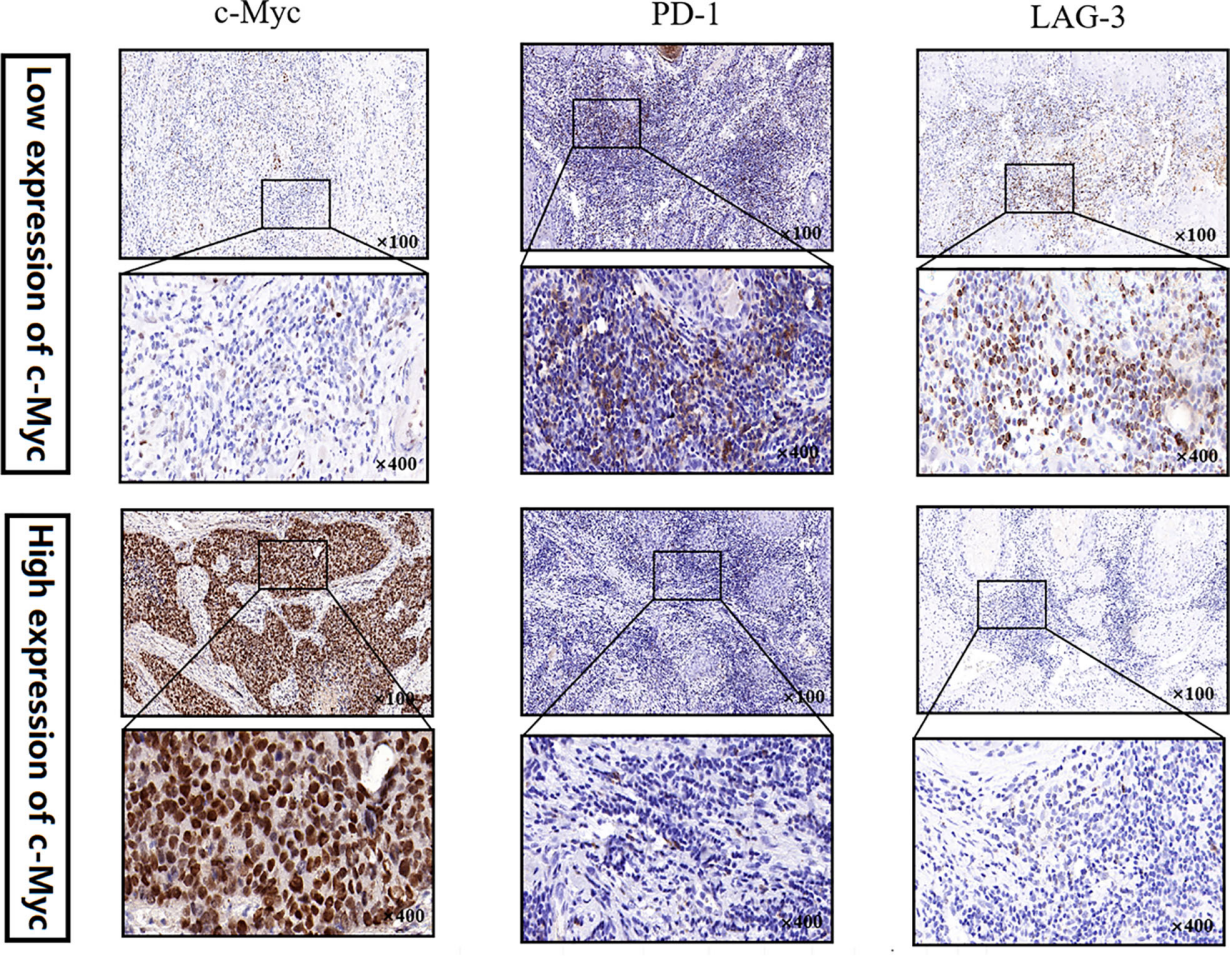
c-Myc, PDCD1 and LAG3 expression in an OSCC TMA by immunohistochemistry.

No significant relationship was found between c-Myc expression and gender, age, tumor staging, and differentiation (**Table 1**). LAG3 protein was expressed on the tumor-infiltrating lymphocytes (TILs) in 15 out of 20 (75%) samples. 45.0% tissues exhibited PDCD1 immunostaining (**Figure 8**). There was a trend that more LAG3 expression was found in patients with low c-Myc expression (p=0.072). There was no significant relationship between c-Myc and PDCD1 (p=0.653).

**Table 1:** The relationship between c-Myc expression and clinicopathological features of OSCC patients.

	Cases, n (%)	c-Myc expression	c-Myc expression	p value
		low, n (%)	high, n (%)	
All patients	20 (100%)	9 (45%)	11 (55%)	
Gender				0.964
Male	11 (55%)	5 (25%)	6 (30%)	
Female	9 (45%)	4 (20%)	5 (25%)	
Age				0.279
≤60 years	7 (35%)	2 (10%)	5 (25%)	
>60 years	13 (65%)	7 (35%)	6 (30%)	
Smoking				0.582
No	12 (60%)	6 (30%)	6 (30%)	
Yes	8 (40%)	3 (15%)	5 (25%)	
TNM staging				0.888
I-II	7 (35%)	3 (15%)	4 (20%)	
III-IV	13 (65%)	6 (30%)	7 (35%)	
Differentiation				0.369
High	4 (20%)	1 (5%)	3 (15%)	
Medium-Low	16 (80%)	8 (40%)	8 (40%)	

## Discussion

Identifying novel molecular prognostic biomarkers is of great importance for improving outcome of HNSC, which develops as a result of a series of genetic and epigenetic alterations in “cancer genes” (tumor suppressors and oncogenes). c-Myc is mainly involved in apoptosis, cell cycle, metabolism, proliferation, and ribosome biogenesis. The overexpression of c-Myc has been almost invariably linked to tumorigenesis. Our results indicated that c-Myc was highly expressed in TP53-mutated HNSC patients. Additionally, high expression of c-Myc in CAL-27 cells was also detected by Western blot. Subsequently, the prognosis analysis results demonstrated that the expression of c-Myc in HNSC could be an independent predictive factor. As a result, due to the downregulation of c-Myc expression in CAL-27 cells, the percentage of apoptotic cells was increased. c-Myc silencing by either shRNA c-Myc or c-Myc inhibitor (10058-F4) resulted in a dose-dependent reduction in long non-coding RNA SNHG16 levels, which induced cell apoptosis in CAL-27 and TSCCA cells (22).

The tumor-immune microenvironment (TME) in HNSC is immunosuppressive and may play an important role in HNSC progression and treatment resistance (23). Understanding the landscape of the tumor-immune microenvironment is critical for improving the efficacy of current immunotherapies in HNSC. The TME is composed of different subsets of cells, such as T cells, B cells, neutrophils, macrophages, regulatory T (Treg) cells, natural killer (NK) cells and mast cells (24). Tumors often evade host immune surveillance by suppressing cytotoxic T cell function or by activating and expanding immunosuppressive cell populations. It is now clear that c-Myc plays a crucial role in instructing the tumor microenvironment (25, 26). c-Myc regulates the TME through effects on both immunoregulatory proteins and immune effector cells. Our results also showed that c-Myc expression was associated with immune cell infiltration. Enhanced tumor infiltration of CD3+CD4+, CD3+CD8+ T cells and NK cells were observed in the Myc-dependent prostate cancer (MycCaP) tissues after small molecule inhibitors (MYCi) treatment (27). Our results showed that an increase percentage of these tumor-infiltrating immune cells were associated with better OS in HNSC patients. MYC inhibitor treatment may induce immunogenic cell death (ICD), which could activate the immune response in tumors leading to immune cell infiltration (27). Myc inhibitor prodrug (MI3-PD) could reduce M2 macrophages in the tumor microenvironment while sparing M1 antitumor macrophages (28). c-Myc inhibitor JQ1 could block M2 polarization of macrophages *via* S1PR1 (29). Mst1-deficiency may induce the hyperactivation of dendritic cells (DCs) (30). IL-23 was increased in Mst1 -/- dendritic cells (DCs), which also exhibited an increase in c-Myc protein levels. c-Myc inhibitor could downregulated the increased expression of IL-23 observed in Mst1 -/- DCs (30). Upon c-Myc activation, there is an immediate exclusion of T, B and NK cells within the tumor microenvironment *via* IL-23 signaling (25). c-Myc also cooperates with Ras to regulate the regulate the secretion of CCL9 and IL-23 (25), thereby promoting the recruitment of immunosuppressive cells and the exclusion of adaptive T and B cells and innate immune NK cells to facilitate tumor immune escape. IL-23 plays a critical role in enhancing IL-17 production *in vivo* (31). KEGG results showed that IL-17 signaling pathway was actived in c-Myc overexpressed HNSC patients. Th17 cells are maintained and expanded by IL-23 *via* tumor-secreted PGE2 (32). Th17 cells further produce IL-17 interacting with IL-17RA/RC complex on receptor carrying cells to regulate functionality of DCs and create a self-sustaining feedback loop *via* IL-23 (33). Induced c-Myc expression could enhanced tumor infiltration of neutrophils, which play a stimulating role in c-Myc-induced liver tumorigenesis (34). Neutrophils play a key role in mediating tumor angiogenesis, however, the increase of neutrophil infiltration could be suppressed by the inhibitor of angiogenesis (34).

The mutational profile of TP53 was an independent prognostic factor in HNSC (35). TP53 mutations occurred in HNSC with a frequency of 72% in a whole-exome sequencing analyses study (36). Mutant p53 proteins not only lose tumor-suppressive functions but also frequently exert oncogenic gain-of-function (GOF) properties through their ability to modulate gene expression (37). c-Myc acted as a major mediator of mutant p53 GOF in HNSC (38). Mutant p53 gains its function *via* c-Myc activation upon CDK4 phosphorylation in HCC (39). Mutant p53 can regulate the expression of the endogenous c-Myc gene and could sustain active the c-Myc promoters (38, 40). Predictive power of coexpression of mutant p53 and c-Myc proteins in outcome of HNSC is more accurately than what these proteins do individually (41). Mutant p53 not only contributes to c-Myc hyperactivation but also enhances c-Myc protein stability by preventing FBW4A-mediated ubiquitination and degradation in HCC cells (42).

HNSC patients exhibited higher c-Myc expression, and the expression of most immune checkpoint molecules, such as CTLA4, HAVCR2, LAG3, and PDCD1, had negative correlations with c-Myc mRNA levels in HNSC in our study. A trend that more LAG3 protein expression was found in patients with low c-Myc expression (p=0.072) was observed in our study. Depending on the cellular context, the c-Myc oncogene could serve as a positive or negative regulator of immune checkpoint molecules expression (43). Knockdown of c-Myc expression in hepatocellular carcinoma cells exposed to IFN-γ using siRNA assay increased expression of PD-L1 both at mRNA and protein levels (44). The expression of PD-L1 could be mediated by the IFNR/JAK/STAT1/IRF1 pathway, yet c-Myc could suppress the expression of STAT1 both at mRNA level and protein level (44). Up-regulation of c-Myc expression during T-cell priming was inhibited by treatment with I-BET-762, which also led to increased expression of the inhibitory cell-surface receptor LAG3 (45). CTLA4 mRNA was significantly less expressed in lymph nodes chronic lymphocytic leukemia (LN-CLL) cells, and c-Myc mRNA was significantly overexpressed in LN-CLL cells (44). Suppression of CTLA4 in chronic lymphocytic leukemia (CLL) patient samples caused a reduction in the levels of c-Myc messenger RNA and protein (46). These results indicate that there was a relationship between c-Myc and CTLA4. HNSC patients with higher CTLA4 levels had longer OS than those with lower CTLA4 levels (P < 0.001) (47). Altogether, it is still unclear how c-Myc might downregulate the expression of immune checkpoint molecules in HNSC.

HERB database, a high-throughput experiment- and reference-guided database of traditional Chinese medicine, was used to find that c-Myc is the target gene of Tripterygium wilfordii Hook (48). Triptonide is a small molecule monomer extract from the ancient Chinese herb Tripterygium wilfordii Hook, which has historically been used in traditional Chinese medicine to treat rheumatoid arthritis for centuries (49). TN induced nasopharyngeal carcinoma (NPC) cell cycle arrest and apoptosis activation through downregulation of c-Myc (50). Our results also showed that Triptonide regulated c-Myc at the transcriptional level to induce apoptosis of CAL-27 cells. A combination of Triptonide and c-Myc-specific siRNA could induce more apoptotic cells than Triptonide used alone, therefore, we suspected that Triptonide could induce the apoptosis of CAL-27 cells through other signaling pathways, such as ERK/MAPK pathway (51).

Several conceptual and practical difficulties, including the lack of defined “pockets” and potential toxicity to normal tissues have led to c-Myc being difficult to target (52). Recent strategies of targeting c-Myc indirectly, such as with BRD4 or CDK7 inhibitors showed a therapeutic window for targeting c-Myc, alleviating the above concerns (53, 54).Han et al. developed the c-Myc inhibitors with well tolerability that disrupted MYC/MAX heterodimerization, enhanced c-Myc degradation, and impaired c-Myc-driven gene expression to increase tumor immune cell infiltration, and sensitize tumors to anti-PD1 immunotherapy (27). The development of novel agents to inhibit c-Myc activity should be highly sought after and a very promising approach for applying targeted therapeutic strategies for cancer therapy.

Several study limitations could have affected our results as well. First, specific data on surgery, chemotherapy, radiotherapy, tumor size and other factors were not available to perform subgroup analyses. Second, the study is based on data from public databases, and the quality of data may affect the results. However, we obtained similar results by analyzing multiple databases. Third, it would be more informative to analyze the difference in immune cell infiltration between different c-MYC expression and TP53 mutation status, since TP53 also highly influences the immunogenic profiles. Forth, only CAL-27 cell line was used to justify the results of bioinformatics analysis and the effect on the cell line may be a random effect. Fifth, we chose Triptonide to interfere with c-Myc expression according to HERB database. In fact, targeting c-Myc by genetic ablation or pharmacological inhibitors may be the best choice to verify the role of c-Myc in HNSC. Lastly, we investigated the molecular functions and biological effects of c-Myc in HSCC by “in silico analysis”, which is within a matter of speculation. The molecular functions and biological effects of c-Myc which should be intensively studied in future investigations.

In conclusion, the current study showed that c-Myc can be considered a therapeutic target for HNSC. However, further functional studies are required to clarify the role of c-Myc in HNSC.

## Data availability statement

The original contributions presented in the study are included in the article/**Supplementary Material**. Further inquiries can be directed to the corresponding authors.

## Ethics statement

The studies involving human participants were reviewed and approved by institutional review board of Nanjing Stomatological Hospital. The patients/participants provided their written informed consent to participate in this study.

## Author contributions

All authors made a significant contribution to the work reported, whether that is in the conception, study design, execution, acquisition of data, analysis and interpretation, or in all these areas; took part in drafting, revising or critically reviewing the article; gave final approval of the version to be published; have agreed on the journal to which the article has been submitted; and agree to be accountable for all aspects of the work.

## Funding

Jiangsu Provincial traditional Chinese medicine science and technology development project, NO. 2021041. Nanjing special fundation for health science and technology development project, NO.ZKX21056.

## Acknowledgments

Thanks are due to Dr. Jing Wang (ORCID 0000-0001-9981-5530) for assistance with the bioinformatic analysis.

## Conflict of interest

The authors declare that the research was conducted in the absence of any commercial or financial relationships that could be construed as a potential conflict of interest.

## Publisher’s note

All claims expressed in this article are solely those of the authors and do not necessarily represent those of their affiliated organizations, or those of the publisher, the editors and the reviewers. Any product that may be evaluated in this article, or claim that may be made by its manufacturer, is not guaranteed or endorsed by the publisher.

## References

[1] Bray F Ferlay J Soerjomataram I Siegel RL Torre LA Jemal A. Global cancer statistics 2018: GLOBOCAN estimates of incidence and mortality worldwide for 36 cancers in 185 countries. CA Cancer J Clin (2018) 68(6):394–424. doi: 10.3322/caac.21492 30207593

[2] Pulte D Brenner H. Changes in survival in head and neck cancers in the late 20th and early 21st century: A period analysis. Oncologist (2010) 15(9):994–1001. doi: 10.1634/theoncologist.2009-0289 20798198 PMC3228039

[3] León X Hitt R Constenla M Rocca A Stupp R Kovács AF. A retrospective analysis of the outcome of patients with recurrent and/or metastatic squamous cell carcinoma of the head and neck refractory to a platinum-based chemotherapy. Clin Oncol (R Coll Radiol) (2005) 17(6):418–24. doi: 10.1016/j.clon.2005.02.014 16149284

[4] Yi L Wu G Guo L Zou X Huang P. Comprehensive analysis of the PD-L1 and immune infiltrates of m6A RNA methylation regulators in head and neck squamous cell carcinoma. Mol Ther Nucleic Acids (2020) 21:299–314. doi: 10.1016/j.omtn.2020.06.001 32622331 PMC7332506

[5] Deng X Jiang Q Liu Z Chen W. Clinical significance of an m6A reader gene, IGF2BP2, in head and neck squamous cell carcinoma. Front Mol Biosci (2020) 7:68. doi: 10.3389/fmolb.2020.00068 32391379 PMC7193208

[6] Sakai A Ando M Fukusumi T Ren S Liu C Qualliotine J. Aberrant expression of CPSF1 promotes head and neck squamous cell carcinoma *via* regulating alternative splicing. PloS One (2020) 15(5):e0233380. doi: 10.1371/journal.pone.0233380 32437477 PMC7241804

[7] Croce CM Thierfelder W Erikson J Nishikura K Finan J Lenoir GM. Transcriptional activation of an unrearranged and untranslocated c-myc oncogene by translocation of a c lambda locus in burkitt. Proc Natl Acad Sci U S A (1983) 80(22):6922–6. doi: 10.1073/pnas.80.22.6922 PMC390098 6417658

[8] Baltaci E Karaman E Dalay N Buyru N. Analysıs of gene copy number changes ın head and neck cancer. Clin Otolaryngol (2018) 43(4):1004–9. doi: 10.1111/coa.12686 27259694

[9] Bhattacharya N Roy A Roy B Roychoudhury S Panda CK. MYC gene amplification reveals clinical association with head and neck squamous cell carcinoma in Indian patients. J Oral Pathol Med (2009) 38(10):759–63. doi: 10.1111/j.1600-0714.2009.00781.x 19453846

[10] Ganesan S. MYC, PARP1, and chemoresistance: BIN there, done that? Sci Signal (2011) 4(166):pe15. doi: 10.1126/scisignal.2001946 21447796

[11] Wang WJ Wu SP Liu JB Shi YS Huang X Zhang QB. MYC regulation of CHK1 and CHK2 promotes radioresistance in a stem cell-like population of nasopharyngeal carcinoma cells. Cancer Res (2013) 73(3):1219–31. doi: 10.1158/0008-5472.CAN-12-1408 23269272

[12] Chandrashekar DS Bashel B Balasubramanya SAH Creighton CJ Ponce-Rodriguez I Chakravarthi BVSK. UALCAN: A portal for facilitating tumor subgroup gene expression and survival analyses. Neoplasia (2017) 19(8):649–58. doi: 10.1016/j.neo.2017.05.002 PMC5516091 28732212

[13] Li T Fu J Zeng Z Cohen D Li J Chen Q. TIMER2.0 for analysis of tumor-infiltrating immune cells. Nucleic Acids Res (2020) 48(W1):W509–14. doi: 10.1093/nar/gkaa407 PMC7319575 32442275

[14] Tang Z Li C Kang B Gao G Li C Zhang Z. GEPIA: a web server for cancer and normal gene expression profiling and interactive analyses. Nucleic Acids Res (2017) 45(W1):W98–W102. doi: 10.1093/nar/gkx247 28407145 PMC5570223

[15] Győrffy B. Survival analysis across the entire transcriptome identifies biomarkers with the highest prognostic power in breast cancer. Comput Struct Biotechnol J (2021)19:4101–09. doi: 10.1016/j.csbj.2021.07.014 PMC8339292 34527184

[16] Anaya J. Guide to using OncoLnc, a new TCGA data portal. Figshare (2016). doi: 10.6084/m9.figshare.2991640.v1

[17] Vasaikar SV Straub P Wang J Zhang B. LinkedOmics: analyzing multi-omics data within and across 32 cancer types. Nucleic Acids Res (2018) 46(D1):D956–63. doi: 10.1093/nar/gkx1090 PMC5753188 29136207

[18] Liu CJ Hu FF Xia MX Han L Zhang Q Guo AY. GSCALite: a web server for gene set cancer analysis. Bioinformatics (2018) 34(21):3771–2. doi: 10.1093/bioinformatics/bty411 29790900

[19] Charoentong P Finotello F Angelova M Mayer C Efremova M Rieder D. Pan-cancer immunogenomic analyses reveal genotype-immunophenotype relationships and predictors of response to checkpoint blockade. Cell Rep (2017) 18(1):248–62. doi: 10.1016/j.celrep.2016.12.019 28052254

[20] Wang J Zhou M Xu JY Chen B Ouyang J. Combination of BCL-2 and MYC protein expression improves high-risk stratification in diffuse large b-cell lymphoma. Oncol Targets Ther (2015) 8:2645–50. doi: 10.2147/OTT.S86093 PMC4583112 26425100

[21] Xu J Shen D Zhang T Wang J De W Zhang J. Lymphocyte-activated gene-3 (LAG3) protein expressed in tumor-infiltrating lymphocytes of colorectal cancer. Pol J Pathol (2021) 72(4):324–30. doi: 10.5114/pjp.2021.114177 35308003

[22] Li S Zhang S Chen J. C-myc induced upregulation of long non-coding RNA SNHG16 enhances progression and carcinogenesis in oral squamous cell carcinoma. Cancer Gene Ther (2019) 26(11-12):400–10. doi: 10.1038/s41417-018-0072-8 30607006

[23] Desrichard A Kuo F Chowell D Lee KW Riaz N Wong RJ. Tobacco smoking-associated alterations in the immune microenvironment of squamous cell carcinomas. J Natl Cancer Inst (2018) 110(12):1386–92. doi: 10.1093/jnci/djy060 PMC6292793 29659925

[24] Kim J Bae JS. Tumor-associated macrophages and neutrophils in tumor microenvironment. Mediators Inflamm (2016) 2016:6058147. doi: 10.1155/2016/6058147 26966341 PMC4757693

[25] Kortlever RM Sodir NM Wilson CH Burkhart DL Pellegrinet L Brown Swigart L. Myc cooperates with ras by programming inflammation and immune suppression. Cell (2017) 171(6):1301–1315.e14. doi: 10.1016/j.cell.2017.11.013 29195074 PMC5720393

[26] Casacuberta-Serra S Soucek L. Myc and ras, the bonnie and Clyde of immune evasion. Transl Cancer Res (2018) 7(Suppl 4):S457–9. doi: 10.21037/tcr.2018.03.09 PMC6774775 31579305

[27] Han H Jain AD Truica MI Izquierdo-Ferrer J Anker JF Lysy B. Small-molecule MYC inhibitors suppress tumor growth and enhance immunotherapy. Cancer Cell (2019) 36(5):483–97. doi: 10.1016/j.ccell.2019.10.001 PMC6939458 31679823

[28] Esser AK Ross MH Fontana F Su X Gabay A Fox GC. Nanotherapy delivery of c-myc inhibitor targets protumor macrophages and preserves antitumor macrophages in breast cancer. Theranostics (2020) 10(17):7510–26. doi: 10.7150/thno.44523 PMC7359087 32685002

[29] Zhou X Liu X Yang X Wang L Hong Y Lian K. Tumor progress intercept by intervening in caveolin-1 related intercellular communication *via* ROS-sensitive c-myc targeting therapy. Biomaterials (2021) 275:120958. doi: 10.1016/j.biomaterials.2021.120958 34130142

[30] Cho KM Kim MS Jung HJ Choi EJ Kim TS. Mst1-deficiency induces hyperactivation of monocyte-derived dendritic cells *via* Akt1/c-myc pathway. Front Immunol (2019) 10:2142. doi: 10.3389/fimmu.2019.02142 31572367 PMC6749027

[31] Stritesky GL Yeh N Kaplan MH. IL-23 promotes maintenance but not commitment to the Th17 lineage. J Immunol (2008) 181(9):5948–55. doi: 10.4049/jimmunol.181.9.5948 PMC2678905 18941183

[32] Qian X Gu L Ning H Zhang Y Hsueh EC Fu M. Increased Th17 cells in the tumor microenvironment is mediated by IL-23 *via* tumor-secreted prostaglandin E2. J Immunol (2013) 190(11):5894–902. doi: 10.4049/jimmunol.1203141 PMC3660540 23645882

[33] Bunte K Beikler T. Th17 cells and the IL-23/IL-17 axis in the pathogenesis of periodontitis and immune-mediated inflammatory diseases. Int J Mol Sci (2019) 20(14):3394. doi: 10.3390/ijms20143394 PMC6679067 31295952

[34] Zhao Y Huang X Ding TW Gong Z. Enhanced angiogenesis, hypoxia and neutrophil recruitment during myc-induced liver tumorigenesis in zebrafish. Sci Rep (2016) 6:31952. doi: 10.1038/srep31952 27549025 PMC4994033

[35] Caponio VCA Troiano G Adipietro I Zhurakivska K Arena C Mangieri D. Computational analysis of TP53 mutational landscape unveils key prognostic signatures and distinct pathobiological pathways in head and neck squamous cell cancer. Br J Cancer (2020) 123(8):1302–14. doi: 10.1038/s41416-020-0984-6 PMC7553957 32684626

[36] Cancer Genome Atlas Network. Comprehensive genomic characterization of head and neck squamous cell carcinomas. Nature (2015) 517(7536):576–82. doi: 10.1038/nature14129 PMC4311405 25631445

[37] Oren M Rotter V. Mutant p53 gain-of-function in cancer. Cold Spring Harb Perspect Biol (2010) 2(2)::a001107. doi: 10.1101/cshperspect.a001107 20182618 PMC2828285

[38] Ganci F Pulito C Valsoni S Sacconi A Turco C Vahabi M. PI3K inhibitors curtail MYC-dependent mutant p53 gain-of-Function in head and neck squamous cell carcinoma. Clin Cancer Res (2020) 26(12):2956–71. doi: 10.1158/1078-0432.CCR-19-2485 31969334

[39] Liao P Zeng SX Zhou X Chen T Zhou F Cao B. Mutant p53 gains its function *via* c-myc activation upon CDK4 phosphorylation at serine 249 and consequent PIN1 binding. Mol Cell (2017) 68(6):1134–46. doi: 10.1016/j.molcel.2017.11.006 PMC6204219 29225033

[40] Frazier MW He X Wang J Gu Z Cleveland JL Zambetti GP. Activation of c-myc gene expression by tumor-derived p53 mutants requires a discrete c-terminal domain. Mol Cell Biol (1998) 18(7):3735–43. doi: 10.1128/MCB.18.7.3735 PMC108956 9632756

[41] Waitzberg AF Nonogaki S Nishimoto IN Kowalski LP Miguel RE Brentani RR. Clinical significance of c-myc and p53 expression in head and neck squamous cell carcinomas. Cancer Detect Prev (2004) 28(3):178–86. doi: 10.1016/j.cdp.2004.02.003 15225897

[42] Wang H Liao P Zeng SX Lu H. Co-Targeting p53-R249S and CDK4 synergistically suppresses survival of hepatocellular carcinoma cells. Cancer Biol Ther (2020) 21(3):269–77. doi: 10.1080/15384047.2019.1685289 PMC7012101 31747859

[43] Glorieux C Xia X Huang P. The role of oncogenes and redox signaling in the regulation of PD-L1 in cancer. Cancers (Basel) (2021) 13(17):4426. doi: 10.3390/cancers13174426 34503236 PMC8431622

[44] Zou J Zhuang M Yu X Li N Mao R Wang Z. MYC inhibition increases PD-L1 expression induced by IFN-γ in hepatocellular carcinoma cells. Mol Immunol (2018) 101:203–9. doi: 10.1016/j.molimm.2018.07.006 30007230

[45] Bandukwala HS Gagnon J Togher S Greenbaum JA Lamperti ED Parr NJ. Selective inhibition of CD4+ T-cell cytokine production and autoimmunity by BET protein and c-myc inhibitors. Proc Natl Acad Sci U S A (2012) 109(36):14532–7. doi: 10.1073/pnas.1212264109 PMC3437860 22912406

[46] Mittal AK Chaturvedi NK Rohlfsen RA Gupta P Joshi AD Hegde GV. Role of CTLA4 in the proliferation and survival of chronic lymphocytic leukemia. PloS One (2013) 8(8):e70352. doi: 10.1371/journal.pone.0070352 23936412 PMC3731360

[47] Liu JN Kong XS Huang T Wang R Li W Chen QF. Clinical implications of aberrant PD-1 and CTLA4 expression for cancer immunity and prognosis: A pan-cancer study. Front Immunol (2020) 11:2048. doi: 10.3389/fimmu.2020.02048 33072070 PMC7539667

[48] Fang S Dong L Liu L Guo J Zhao L Zhang J. HERB: a high-throughput experiment- and reference-guided database of traditional Chinese medicine. Nucleic Acids Res (2021) 49(D1):D1197–206. doi: 10.1093/nar/gkaa1063 PMC7779036 33264402

[49] Leuenroth SJ Okuhara D Shotwell JD Markowitz GS Yu Z Somlo S. Triptolide is a traditional Chinese medicine-derived inhibitor of polycystic kidney disease. Proc Natl Acad Sci U S A (2007) 104(11):4389–94. doi: 10.1073/pnas.0700499104 PMC1838612 17360534

[50] Wang SS Lv Y Xu XC Zuo Y Song Y Wu GP. Triptonide inhibits human nasopharyngeal carcinoma cell growth *via* disrupting lnc-RNA THOR-IGF2BP1 signaling. Cancer Lett (2019) 443:13–24. doi: 10.1016/j.canlet.2018.11.028 30503558

[51] Zheng L Fang S Hui J Rajamanickam V Chen M Weng Q. Triptonide modulates MAPK signaling pathways and exerts anticancer effects *via* ER stress-mediated apoptosis induction in human osteosarcoma cells. Cancer Manag Res (2020) 12:5919–29. doi: 10.2147/CMAR.S258203 PMC7373419 32765093

[52] McKeown MR Bradner JE. Therapeutic strategies to inhibit MYC. Cold Spring Harb Perspect Med (2014) 4(10):a014266. doi: 10.1101/cshperspect.a014266 25274755 PMC4200208

[53] Yin M Guo Y Hu R Cai WL Li Y Pei S. Potent BRD4 inhibitor suppresses cancer cell-macrophage interaction. Nat Commun (2020) 11(1):1833. doi: 10.1038/s41467-020-15290-0 32286255 PMC7156724

[54] Wang J Zhang R Lin Z Zhang S Chen Y Tang J. CDK7 inhibitor THZ1 enhances antiPD-1 therapy efficacy *via* the p38α/MYC/PD-L1 signaling in non-small cell lung cancer. J Hematol Oncol (2020) 13(1):99. doi: 10.1186/s13045-020-00926-x 32690037 PMC7370470

