# Supplementary material for: Identification and validation of the role of c-Myc in head and neck squamous cell carcinoma
Source: Front Oncol. 2022 Aug 31;12:820587. doi: 10.3389/fonc.2022.820587 (PMC9470836; doi:10.3389/fonc.2022.820587)
Supplement: Supplementary file 1 [file DataSheet_1.doc]

***1.apoptosis***


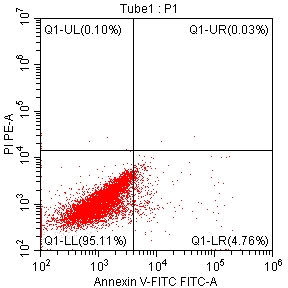


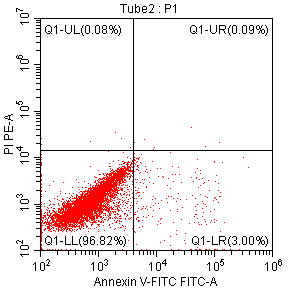


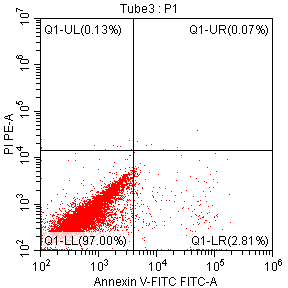


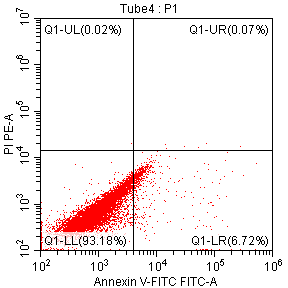


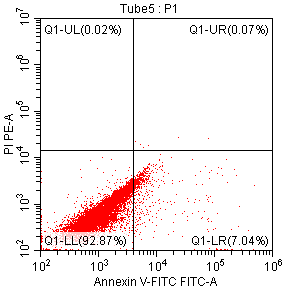


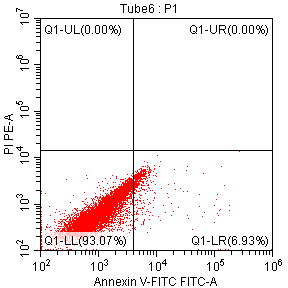


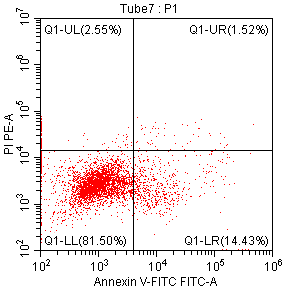

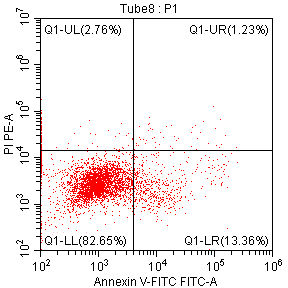


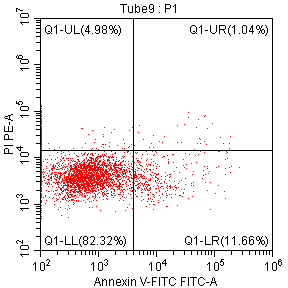


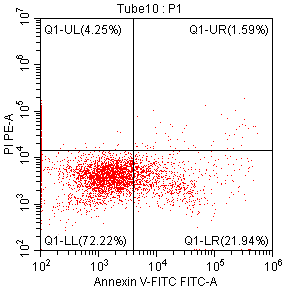

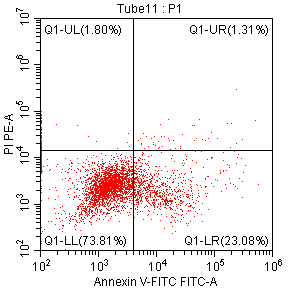


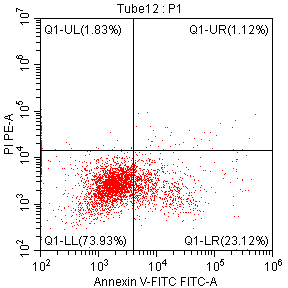


| **Group** | **UL(%)** | **UR(%)** | **LL(%)** | **LR(%)** | **Apoptosis(%)** |
| --- | --- | --- | --- | --- | --- |
| CON | 0.10 | 0.03 | 95.11 | 4.76 | 4.79 |
|  | 0.08 | 0.09 | 96.82 | 3.00 | 3.09 |
|  | 0.13 | 0.07 | 97.00 | 2.81 | 2.88 |
| 10nM | 0.02 | 0.07 | 93.18 | 6.72 | 6.79 |
|  | 0.02 | 0.07 | 92.87 | 7.04 | 7.11 |
|  | 0.00 | 0.00 | 93.07 | 6.93 | 6.93 |
| 30nM | 2.55 | 1.52 | 81.50 | 14.43 | 15.95 |
|  | 2.76 | 1.23 | 82.65 | 13.36 | 14.59 |
|  | 4.98 | 1.04 | 82.32 | 11.66 | 12.70 |
| 50nM | 4.25 | 1.59 | 72.22 | 21.94 | 23.53 |
|  | 1.80 | 1.31 | 73.81 | 23.08 | 24.39 |
|  | 1.83 | 1.12 | 73.93 | 23.12 | 24.24 |

***Western blot***


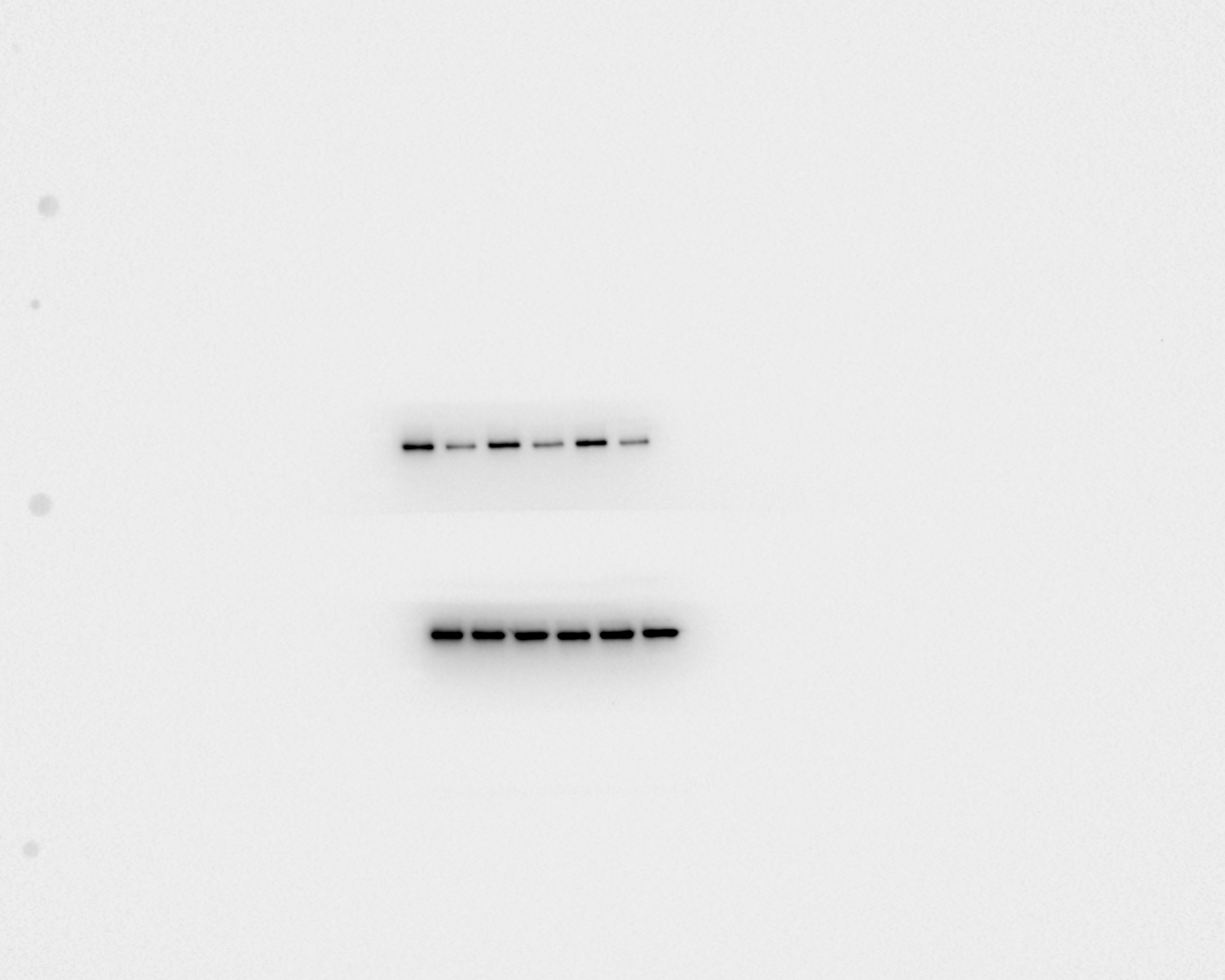


| GROUP | control | triptonide 50nM | control | triptonide 50nM | control | triptonide 50nM |
| --- | --- | --- | --- | --- | --- | --- |
| MYC | 1730.8 | 386.18 | 1519.9 | 392.84 | 1653.5 | 341.07 |
| GAPDH | 3380.2 | 3295.2 | 3316.3 | 3184 | 3472.7 | 3393.3 |
|  |  |  |  |  |  |  |
|  |  |  |  |  |  |  |
|  |  |  |  |  |  |  |
| GROUP | control | triptonide 50nM | control | triptonide 50nM | control | triptonide 50nM |
| MYC/GAPDH | 0.51 | 0.12 | 0.46 | 0.12 | 0.48 | 0.10 |

***RT-PCR***


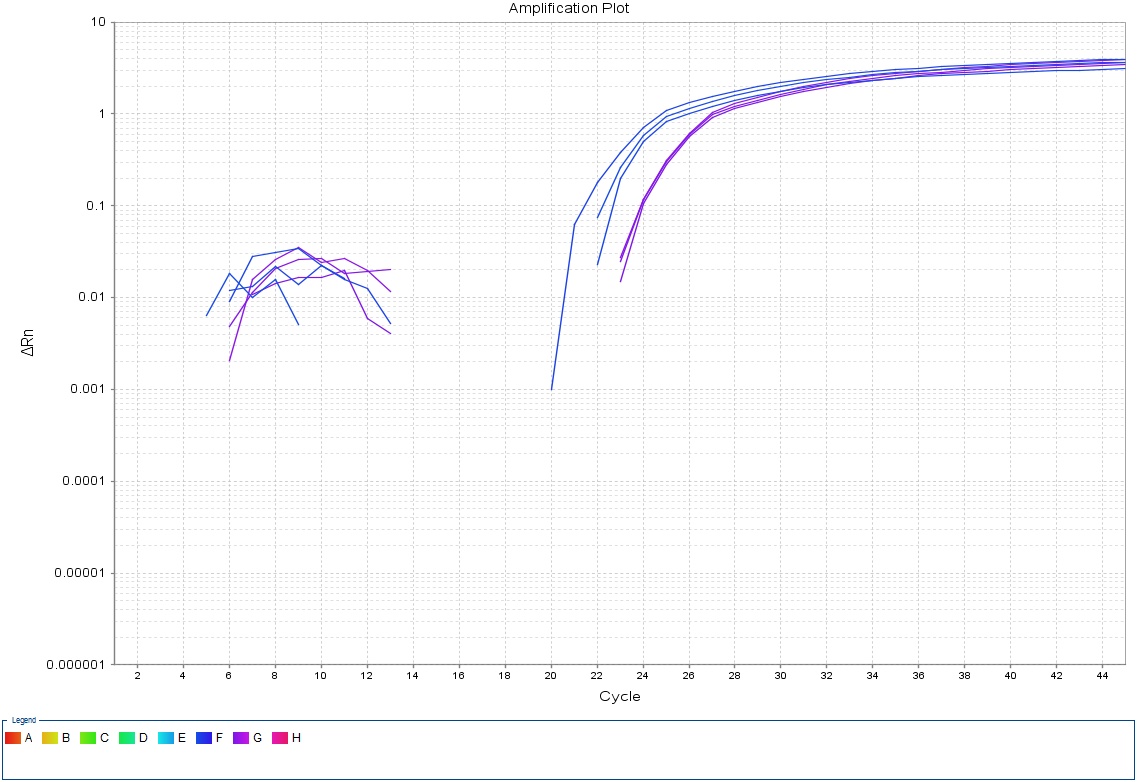


| Sample | MYC |
| --- | --- |
| control | 1 |
| triptonide50nM | 0.5±0.05 |
